# If the first child is breech, overall outcomes for families with two children are similar regardless of the mode of the first birth

**DOI:** 10.1038/s41598-024-76433-7

**Published:** 2024-10-16

**Authors:** Julia Savchenko, Cecilia Pegelow Halvorsen, Pelle G Lindqvist, Sophia Brismar Wendel

**Affiliations:** 1 Department of Obstetrics and Gynecology, Stockholm South General Hospital (Södersjukhuset), Stockholm, Sweden; 2 Department of Clinical Science and Education, Stockholm South General Hospital (Södersjukhuset), Karolinska Institutet, Stockholm, Sweden; 3 Neonatal unit, Sachs’ Children and Youth Hospital, Södersjukhuset, Stockholm, Sweden; 4 Department of Obstetrics and Gynecology, Danderyd Hospital, Stockholm, Sweden; 5 Department of Clinical Sciences, Danderyd Hospital, Karolinska Institutet, Stockholm, Sweden

**Keywords:** Breech birth, Vaginal birth, Cesarean section, Adverse perinatal outcome, Birth after cesarean section, Health care, Medical research

## Abstract

Cesarean section for breech presentation is often recommended. However, cesarean section affects future reproduction. The aim of this study was to assess the effect of mode of the first birth in breech on outcomes of the second birth and the two births together. This is a register-based nationwide cohort study including 23 062 women with a first singleton birth in breech ≥ 34 gestational weeks and a subsequent singleton birth in Sweden 2000–2019. Exposure was mode of first delivery. Main maternal outcome was a composite of fourth-degree perineal injury, postpartum hemorrhage requiring blood transfusion, hysterectomy, or death. Main infant outcome was a composite of stillbirth, extremely preterm birth (< 28 weeks), moderate to severe hypoxic ischemic encephalopathy, therapeutic hypothermia, or death. Outcomes were analyzed using multivariable logistic regression. In the first birth, the infant composite outcome affected < 1% in both groups but the risk was higher in the vaginal breech group (13/1525), compared with the breech CS group (27/21 537), aOR 7.06, 95% CI 2.91–17.1. In the second birth, the infant composite outcome affected < 1% in both groups but the risk was lower for the first vaginal breech group (3/1525) compared with the first breech CS group (152/21 537), aOR 0.26, 95% CI 0.08–0.84. There was no significant difference between the groups in risk of composite infant outcome in the two births assessed together, or in risk of composite maternal outcome. In total, the chance of a two-children family without maternal or infant severe adverse composite outcome was high and similar regardless mode of the breech first birth.

**Supplementary Information:**

The online version contains supplementary material available at 10.1038/s41598-024-76433-7.

## Introduction

Vaginal breech birth has been associated with increased short-term perinatal mortality and neonatal morbidity compared with planned breech cesarean section (CS)^1–3^. To avoid these risks, planned CS is often recommended as mode of delivery for persistent breech presentation^4,5^ However, planned breech CS involves surgical risks with increased short-term maternal morbidity and may also impact long-term infant health^1,3,6^ CS may also impact future health and outcomes at subsequent pregnancies including the risk of repeat cesarean section, placenta accreta spectrum disorders, hysterectomy, and uterine rupture with associated neonatal morbidity^7–10^.

According to a Cochrane systematic review on planned CS for term breech delivery, “the benefits need to be weighed against factors such as the mother’s preference for vaginal birth and risks such as future pregnancy complications in the woman’s specific healthcare setting”^1^. Due to the complex combination of risks for the mother and child, the Royal College of Obstetricians and Gynaecologists Green-top Guideline on management of breech presentation states that “women should be informed that cesarean section increases the risk of complications in future pregnancy”, and “clinicians should counsel women in an unbiased way that ensures a proper understanding of the absolute as well as relative risks of their different options”^5^. This viewpoint is shared by other professional associations^4,11,12^ Breech as indication for first CS has been reported to be a predictor for higher success of vaginal birth after cesarean^13,14^ However, there is limited data on the absolute and relative risks for future siblings of children born in breech presentation, as well as for mothers pregnant after previous breech CS or vaginal breech birth^15–17^. Recommendations regarding absolute and relative risks have therefore mostly relied on indirect evidence combining results from studies on breech birth^1^, benefits of a previous vaginal delivery^18–20^, and risks of repeat cesarean section and trial of labor after cesarean for all indications^7–10^.

To facilitate counselling and informed choice in this multifactorial complex situation, we performed this nationwide linked register study with the objective to assess the effect of mode of delivery of the first child in breech presentation on maternal and infant outcomes in the first and second birth separately, as well as on the total outcome burden after the two births.

## Materials and methods

Data was extracted from the Swedish Medical Birth Register and linked with data from Statistics Sweden, the Cause of Death Register, the National Patient Register, and the Swedish Neonatal Quality Register (SNQ) using Swedish personal identification numbers unique to all individuals in Sweden. The Swedish Medical Birth Register, held by the National Board of Health and Welfare, is mandatory, validated, and contains prospectively collected data on 98% of all births ≥ 22 gestational weeks, including demographics, reproductive history, maternal diseases, and pregnancy complications classified using the Swedish version of the International Classification of Diseases 10th edition (ICD-10)^21^. The Cause of Death Register held by the National Board of Health and Welfare is also a mandatory register containing diagnostic codes determining numbered causes of death and has been validated with satisfactory results for certain outcomes relevant for this study^22^. The National Patient Register held by the National Board of Health and Welfare covers 99% of inpatient and specialized outpatient care, including diagnostic and treatment codes and timepoints of care^23^. The SNQ is a quality register containing detailed data on neonatal inpatient care with 98–99% coverage depending on gestational age^24^.

The study population was limited to all women with a first and a second birth in Sweden from 2000 to 2019, whereof the first birth was a singleton birth in breech presentation at 34 gestational weeks or more. The study period was chosen based on the considerable change in management of breech presentation after the Term Breech Trial^25^, until most recent available data. Women with antepartum stillbirth in the first birth or multiple birth in the first or second birth were excluded because of special obstetric management strategies in these cases.

Populational characteristics before or at the first birth were described in numbers and proportions including age (categorized in < 20, 20–24, 25–29, 30–34, 35–39, and ≥ 40 years), height (categorized in ≤ 160, 161–170, and > 170 cm), body mass index (BMI) at the first antenatal visit (categorized in < 18.5, 18.5–24.9, 25.0–29.9, 30–34.9, and ≥ 35.0 kg/m^2^), country of birth (categorized as Sweden, Europe/North America, other, or unknown), history of infertility or in vitro fertilization (based on marked checkboxes), hypertensive disease before pregnancy (based on ICD codes I10–I15, O10 and marked checkbox for previous hypertension), hypertensive complications in pregnancy (based on ICD codes O11, O13–O16), placental complications defined as placenta previa (ICD code O44), placenta accreta (ICD code O432), or placental abruption (ICD code O45), mode of labor onset (based on marked checkboxes for spontaneous, induction, or prelabor cesarean, and ICD codes O61 and O756B for labor induction, O756A for spontaneous start), gestational week (categorized in 34–36, 37–38, 39–40, 41, ≥ 42 complete weeks, or unknown), birthweight (categorized in < 1000, 1000–1499, 1500–2499, 2500–3999, 4000–4499, ≥ 4500 g, or unknown), year of birth (categorized in 2000–2004, 2005–2009, 2010–2014, and 2015–2019), and presence of fetal central nervous system malformations or chromosomal abnormality (ICD codes Q00–Q07, Q90–Q99 or marked checkboxes in SNQ), maternal education (categorized in compulsory school or less, upper secondary school, college or university ≤ 2 years, college or university > 2 years, or unknown), smoking in pregnancy (categorized in yes, no, and unknown, based on marked checkboxes), pregestational diabetes (based on marked checkbox and ICD codes E10–E11 and O240–O243), or gestational diabetes (based on ICD code O244 or O249), and fetal sex (male, female, unknown) were collected.

The main maternal outcome measure was a composite of severe adverse outcomes including at least one of the following: fourth-degree perineal injury (ICD code O703 or a marked checkbox indicating injury to the rectum), severe postpartum hemorrhage (> 1000 ml) requiring blood transfusion (ICD code O72 together with treatment code DR029), hysterectomy (ICD code O822 or treatment code LCD), or death within one year postpartum. Secondary maternal outcomes were any of the components of the composite outcome, obstetric anal sphincter injury (OASI, including third- or fourth-degree perineal injury based on ICD codes O702 or O703, treatment code MBC33, or marked checkboxes indicating injury to the sphincter or rectum), and for the second birth placental complications (as defined above), uterine rupture (ICD codes O710–O711), and mode of delivery. Missing data were categorized as “unknown” wherever possible to avoid exclusion of these women in the multivariable regression analyses.

The main infant outcome measure was a composite adverse outcome including at least one of the following: stillbirth, extremely preterm birth (< 28 gestational weeks), moderate to severe hypoxic ischemic encephalopathy (defined as ICD codes P916B or P916B, or marked checkbox in SNQ), therapeutic hypothermia (defined as treatment code DV034), or death within one year after birth. Secondary infant outcomes were any of the components of the composite outcome, Apgar score < 4 at 5 min, Apgar score < 6 at 10 min, and for the second birth very preterm birth (< 32 gestational weeks) and preterm birth (< 37 gestational weeks).

A total outcome burden for the first and second birth was described, including composite severe maternal outcome in any of the two births, composite severe maternal outcome in both births, composite severe infant outcome in any of the two births, composite severe infant outcome in both births, and “happy family”: neither maternal nor infant composite severe outcome in any of the two births.

### Statistical analyses

**Fig. 1:**
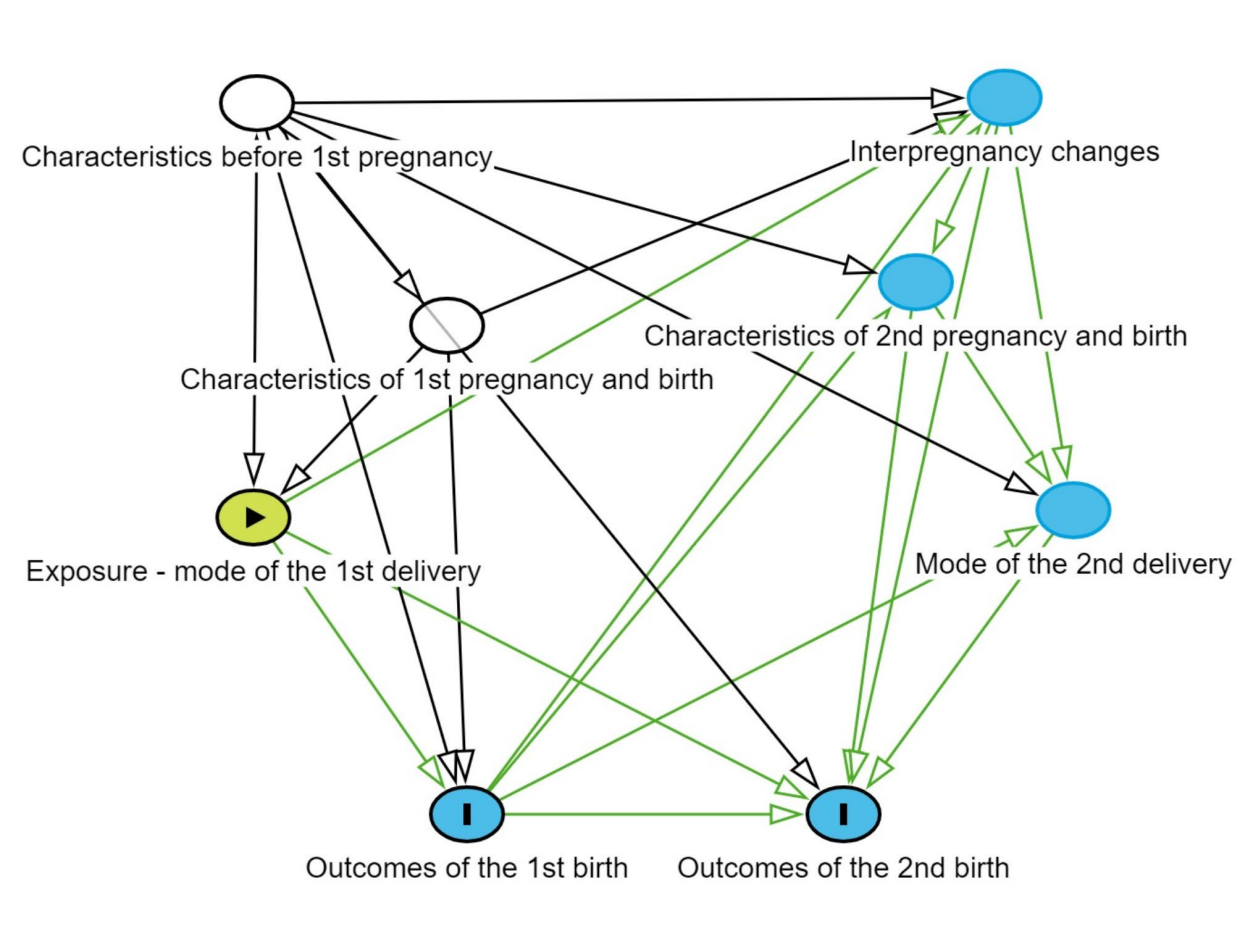
Directed acyclic graph for the study.

The cohort of women was divided in two groups: Women with a first vaginal breech birth and women with a first breech CS. Populational characteristics before and at first birth were compared between the two groups using Pearson Chi^2^ test or Fisher’s exact test for numbers below five. Outcomes of the first and second births are described in numbers and proportions. The risks of these outcomes for the first vaginal breech birth group were estimated with univariable and multivariable logistic regression using the first cesarean breech birth group as the reference group. The risks are presented as crude and adjusted odds ratios (aOR) with 95% confidence intervals (CI). Directed acyclic graphs were drawn to select variables for the multivariable regression model using www.dagitty.net (Fig. 1). Characteristics that differed between the groups with *P* ≤ 0.10 were interpreted as possible confounders. Gestational age and birthweight were considered to interact considerably, and this interaction term was used. The final model included following characteristics before or at the first birth: maternal age, height, BMI, country of birth, infertility, or in vitro fertilization (IVF), preeclampsia/pregnancy related hypertension, placental complications, labor onset, year of birth, gestational age at birth, birthweight, and presence of fetal central nervous system malformations or chromosomal abnormality, categorized as described above. A large proportion of reported congenital CNS malformations in Sweden involve minor conditions diagnosed after birth, while most pregnancies with prenatally diagnosed chromosomal abnormalities are terminated. Furthermore, it was in our data impossible to distinguish placental complications occurring before labor from those identified intrapartum. Therefore, we decided not to exclude these two groups from the study population. Instead, we adjusted for these variables in the regression model. In addition, sensitivity analyses were performed by assessing the main outcomes excluding placental complications, fetal CNS malformations, and chromosomal abnormalities. IBM SPSS Statistics for Windows, Version 28.0.1.1 (Armonk, NY, USA) was used for the statistical analyses.

We report the results according to the STROBE statement (supporting information, Table S1)^26^. A core outcome set is reported according to the Swedish Perinatal Core Outcome Set (SPeCOS) recommendation (supporting information, Tables S2 and S3)^27^. Patients were not involved in the design of this study but contributed to the choice of outcomes through their involvement in the development of the SPeCOS recommendation^27^.

### Ethics Statement

Ethics approval was obtained prior to study start from the Swedish Ethical Review Authority on January 7, 2021 (reference number 2019–05458), with the amendment approved on August 6, 2021 (reference number 2021–02706). Research was conducted in accordance with relevant guidelines and regulations for a restrospective study analyzing pseudonymized register data. Informed patient consent is not required for data retrieval from mandatory registers according to Swedish legislation.

## Results

**Fig. 2:**
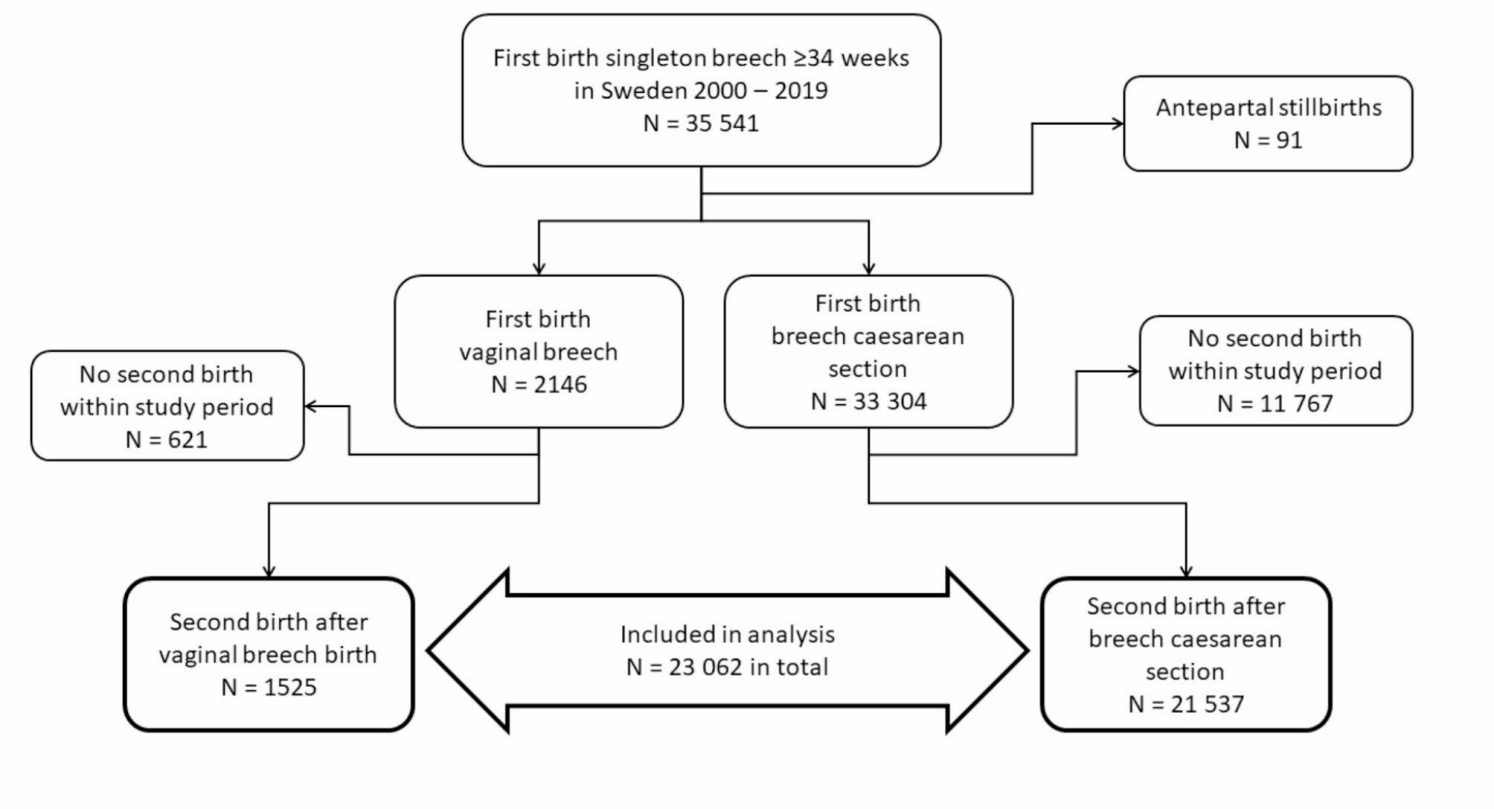
Flow chart of the study.

During the study period, 35 541 women had a first singleton birth in breech at 34 gestational weeks or more. Of these, 91 (0.3%) had an antepartum stillbirth and were excluded, leaving 2146 (6.0%) women with a first vaginal breech birth and 33 304 (93.7%) women with first breech CS. Of these, 1525 (71.1%) women with a previous breech vaginal delivery and 21 537 (64.7%) women with a previous breech CS had a second child (*P* < 0.001). These 23 062 women fulfilled eligibility criteria and were included (Fig. 2).

The women in the first vaginal breech group were somewhat younger, taller, slimmer, more often born in Sweden, less often with a history of infertility or IVF, hypertensive disease, or placental complications, more often gave birth before or after term, earlier in the study period, and to a child of lower birthweight, than women in the first breech CS group (Table 1). There was no significant difference in maternal education, smoking, hypertension before pregnancy, pregestational or gestational diabetes, or fetal sex (not in Tables).

**Table 1:** Populational characteristics before or at the first birth in breech ≥ 34 gestational weeks.

		Vaginal breech *N* = 1525		Cesarean breech *N* = 21 537		*P*
		*n*	%	*n*	%	
Age (years)	< 20	19	1.2	342	1.6	< 0.001
	20–24	270	17.7	3276	15.2	
	25–29	678	44.5	8486	39.4	
	30–34	463	30.4	7540	35.0	
	35–39	89	5.8	1772	8.2	
	≥ 40	6	0.4	121	0.6	
Height (cm)	≤ 160	183	12.0	3582	16.6	< 0.001
	161–170	794	52.1	11 572	53.7	
	> 170	477	31.3	5211	24.2	
	Unknown	71	4.7	1172	5.4	
BMI (kg/m^2^)	< 18.5	43	2.8	511	2.4	< 0.001
	18.5–24.9	990	64.9	13 097	60.8	
	25.0–29.9	261	17.1	4098	19.0	
	30–34.9	53	3.5	1082	5.0	
	≥ 35.0	16	1.0	433	2.0	
	Unknown	162	10.6	2316	10.8	
Place of birth	Sweden	18 338	85.8	1309	85.1	0.023
	Europe, North America	1366	7.5	114	6.3	
	Other	1817	6.6	100	8.4	
	Unknown	16	0.1	2	0.1	
Infertility/IVF		191	12.5	3029	14.1	0.094
Preeclampsia/gestational hypertension		15	1.0	634	2.9	< 0.001
Placental complication		1	0.1	163	0.8	< 0.001
Labor onset	Spontaneous	1475	96.7	5999	27.9	< 0.001
	Induction	37	2.4	163	0.8	
	Prelabor cesarean	0	0.0	14 921	69.3	
	Unknown	13	0.9	454	2.1	
Gestational week	34–36	257	16.9	2123	9.9	< 0.001
	37–38	379	24.9	10 369	48.1	
	39–40	690	45.2	7979	37.0	
	41	166	10.9	798	3.7	
	≥ 42	33	2.2	268	1.2	
Birthweight (g)	< 1000	1	0.1	0	0.0	< 0.001
	1000–1499	1	0.1	16	0.1	
	1500–2499	138	9.0	1326	6.2	
	2500–3999	1343	88.1	18 718	86.9	
	4000–4499	38	2.5	1254	5.8	
	≥ 4500	0	0.0	204	0.9	
	Unknown	4	0.3	19	0.1	
Year of birth	2000–2004	658	43.1	5979	27.8	< 0.001
	2005–2009	382	25.0	6863	31.9	
	2010–2014	330	21.6	6209	28.8	
	2015–2019	155	10.2	2486	11.5	
CNS malformation/chromosomal anomaly		39	2.6	159	0.7	< 0.001

BMI: body mass index, IVF: in vitro fertilization, CNS: central nervous system.

In the first birth, the proportion affected by the maternal composite outcome was < 1% with a similar risk in the two groups (Table 2). The proportion affected by the infant composite outcome was < 1% in both groups but the risk of infant composite outcome was higher in the vaginal breech group (aOR 7.06, 95% CI 2.91–17.1) compared with the breech CS group (Table 2). Among secondary outcomes, the risk of Apgar score < 4 at 5 min, and Apgar score < 6 at 10 min, infant death within one year after birth, as well as the risk of OASI grade 3–4, were higher in the vaginal breech group compared with the breech CS group (Table 2).

**Table 2:** Outcomes of the first birth in breech ≥ 34 gestational weeks.

	Vaginal breech birth *N* = 1525		Breech cesarean section *N* = 21 537		Risk estimates with breech cesarean section as reference		
	*n*	%	*n*	%	OR	aOR^a^	95% CI^a^
Maternal composite outcome	15	0.98	177	0.82	1.20	1.26	0.71–2.24
OASI grade 4	6	0.39	0	0	n/a	n/a	n/a
PPH with blood transfusion	9	0.59	177	0.82	0.72	0.75	0.37–1.53
Hysterectomy	0	0	0	0	n/a	n/a	n/a
Death within one year	0	0	0	0	n/a	n/a	n/a
Infant composite outcome	13	0.85	27	0.13	6.85	7.06	2.91–17.1
Intrapartum stillbirth	2	0.13	0	0	n/a	n/a	n/a
Extreme prematurity (< 28 gw)	n/a	n/a	n/a	n/a	n/a	n/a	n/a
HIE grade 2–3	1	0.07	3	0.01	4.71	9.61	0.36–257
Therapeutic hypothermia	0	0	1	0.00	n/a	n/a	n/a
Death within one year	10	0.66	24	0.11	5.92	6.33	2.44–16.4
Apgar score < 4 at 5 min	8	0.52	8	0.04	14.2	7.63	2.41–24.1
Apgar score < 6 at 10 min	8	0.52	9	0.04	12.6	13.74	3.85–49.0
OASI grade 3–4	63	4.13	1	0.00	928.0	n/a	n/a

OR: odds ratio, aOR: adjusted OR, CI: confidence interval, OASI: obstetric anal sphincter injury, PPH: postpartum hemorrhage, HIE: hypoxic ischemic encephalopathy gw – gestational weeks, n/a: not applicable.

^a^Adjusted for first birth maternal age, height, BMI, origin, infertility history, pregnancy-related hypertensive disorders, placental complications, type of labor onset, year of birth, gestational week, birthweight, and presence of fetal CNS malformations or chromosomal abnormality.

In the second birth, the proportion affected by the maternal composite outcome was < 1% in first vaginal breech birth group and around 1% in first breech CS group, but the difference between groups was not statistically significant (Table 3). The proportion affected by the infant composite outcome was < 1% in both groups but the risk of infant composite outcome was lower for the first vaginal breech group (aOR 0.26, 95% CI 0.08–0.84) compared with the first breech CS group (Table 3). None of the separate maternal or infant outcome components carried a significantly increased risk alone (Table 3). Among the secondary outcomes, there was a decreased risk of OASI grade 3–4, uterine rupture, operative vaginal and cesarean birth, very preterm birth before 32 gestational weeks, and preterm birth before 37 gestational weeks, in the first vaginal breech group compared with the first breech CS group (Table 3). Some of the outcomes (Apgar score < 6 at 10 min, HIE grade 2–3, therapeutic hypothermia, maternal death, placenta accreta) occurred only in the first breech CS group, but the difference did not reach statistical significance (Table 3).

**Table 3:** Outcomes of the second birth by mode of the first birth in breech ≥ 34 gestational weeks.

	Previous vaginal breech birth *N* = 1525		Previous breech cesarean section *N* = 21 537		Risk estimates with previous cesarean section as reference		
	*n*	%	*n*	%	OR	aOR^a^	95% CI^a^
Maternal composite outcome	9	0.59	239	1.11	0.53	0.51	0.25–1.02
OASI grade 4	1	0.07	92	0.43	0.15	0.15	0.02–1.12
PPH requiring blood transfusion	5	0.33	126	0.59	0.56	0.58	0.23–1.49
Hysterectomy within one year	4	0.26	22	0.10	2.57	1.88	0.56–6.31
Death within one year	0	0.00	2	0.01	n/a	n/a	n/a
Infant composite outcome	3	0.20	152	0.71	0.28	0.26	0.08–0.84
Stillbirth	1	0.07	60	0.28	0.24	0.20	0.03–1.53
Extreme prematurity (< 28 gw)	1	0.07	55	0.26	0.26	0.17	0.02–1.29
HIE grade 2–3	0	0.00	23	0.11	n/a	n/a	n/a
Therapeutic hypothermia	0	0.00	22	0.10	n/a	n/a	n/a
Death within 1 year	1	0.07	38	0.18	0.37	0.42	0.05–3.24
Mode of delivery: Spontaneous vaginal	1402	91.93	12 173	56.52	8.77	7.23	5.95–8.77
Operative vaginal	18	1.18	1901	8.83	0.12	0.11	0.07–0.18
Cesarean section	105	6.89	7463	34.65	0.14	0.18	0.15–0.22
OASI grade 3–4	18	1.18	1318	6.12	0.18	0.17	0.11–0.28
Any placental complication	11	0.72	234	1.09	0.66	0.79	0.41–1.51
Placenta previa	1	0.07	100	0.46	0.14	0.17	0.02–1.24
Placenta accreta	0	0	22	0.10	n/a	n/a	n/a
Placental abruption	10	0.66	119	0.55	1.19	1.36	0.66–2.79
Uterine rupture	0	0	209	0.97	n/a	n/a	n/a
Very preterm birth (< 32 gw)	5	0.33	154	0.72	0.46	0.39	0.15–0.97
Preterm birth (< 37 gw)	98	6.43	1161	5.39	1.21	0.76	0.61–0.95
Apgar score < 4 at 5 min	1	0.07	44	0.20	0.32	0.40	0.05–3.11
Apgar score < 6 at 10 min	0	0.00	45	0.21	n/a	n/a	n/a

OR: odds ratio, CI: confidence interval, PPH: postpartum hemorrhage, OASI: obstetric anal sphincter injury, HIE: hypoxic ischemic encephalopathy, gw – gestational weeks, n/a: not applicable.

^a^Adjusted for first birth maternal age, height, BMI, origin, infertility history, pregnancy-related hypertensive disorders, placental complications (previa, accrete or abruption), type of labor onset, year of birth, gestational age at birth, birthweight, and presence of fetal CNS malformations or chromosomal abnormality.

For the two births together, no family was affected by the composite infant outcome in both births, and there was no significant difference in risk of composite maternal outcome in both births, composite maternal outcome in any of the two births, composite infant outcome in any of the two births, or neither outcome in any of the two births (“happy family”) between the first vaginal breech group and the first breech CS group (Table 4).

**Table 4:** Composite maternal and infant outcomes in the first and second birth together, by mode of first birth in breech ≥ 34 gestational weeks.

	First vaginal breech birth *N* = 1525		First breech cesarean section *N* = 21 537		Risk estimates with first cesarean section as reference		
	*n*	%	*n*	%	OR	aOR^a^	95% CI^a^
Maternal outcome^b^ in any of the two births	22	1.44	366	1.70	0.85	0.86	0.54–1.36
Maternal outcome^b^ in both births	2	0.13	50	0.23	0.56	0.50	0.11–2.16
Infant outcome^c^ in any of the two births	16	1.05	179	0.83	1.27	1.19	0.68–2.09
Infant outcome^c^ in both births	0	0	0	0.00	n/a	n/a	n/a
Happy family: neither outcome in any birth	1487	97.51	21 002	97.52	1.00	1.00	0.70–1.43

OR: odds ratio, aOR: adjusted OR, CI: confidence interval.

^a^Adjusted for first birth maternal age, height, BMI, country of birth, infertility/IVF, pregnancy-related hypertensive disorders, placental complications (previa, accreta or abruption), labor onset, year of birth, gestational age, birthweight, and presence of fetal central nervous system or chromosomal anomalies.

^b^Maternal composite adverse outcome: at least one of the following – fourth-degree perineal injury, postpartum hemorrhage requiring blood transfusion, hysterectomy or death within one year postpartum.

^c^Infant composite adverse outcome: at least one of the following – stillbirth, extremely preterm birth (< 28 gestational weeks), moderate to severe hypoxic ischemic encephalopathy, therapeutic hypothermia, or death within one year after birth.

The sensitivity analysis of the main outcomes by excluding women with known placental complications at first birth (previa, accreta or abruption), or infants with fetal central nervous system malformations or chromosomal abnormality, did not change the result (data not presented). The numbers and proportions of the perinatal outcomes according to the Swedish Perinatal Core Outcome Set are reported in the supporting information (Tables S2 and S3).

## Discussion

This nationwide linked register-based study showed low rates of severe maternal and infant outcomes in the first and second birth and similar chance for the mother and her first two children in total to avoid severe maternal and infant outcomes, regardless of if the first child in breech was born vaginally or by CS, i.e., a similar “happy family” rate. The risk of the maternal composite outcome at the first and the second birth were similar between the women with a first vaginal breech and women with a first breech CS. The risk of the infant composite outcome was higher for the first child born vaginally in breech, which was balanced by the reduced risk for its younger sibling.

The main strength of this study is its sample size including over 23 000 women with two consecutive births, combining rich administrative data from national Swedish registers with next to complete coverage^21–24,28^ To the best of our knowledge, this is the first study to assess maternal and infant risks in the first birth in breech and the second birth together, up to one year after delivery, giving a total risk estimate for a family with two children.

Main outcome measures in this study are maternal and infant combined severe adverse outcomes. The components included into the composite outcomes were a priori selected so that they would be unequivocally important for families to avoid. Main infant outcome combines death and conditions associated with significant risk of long-term sequelae, including HIE grade 2–3, need of therapeutic hypothermia, and extremely preterm birth (justified by known association between preterm birth and previous cesarean section and the substantially increased risk of mortality and severe, including long-term, morbidity in persons born extremely preterm, as well as traumatic experience for families)^29–34^. Main maternal outcome includes death and major morbidity, either life-threatening as major postpartum hemorrhage requiring blood transfusion or hysterectomy, or fourth-degree perineal tears, feared by women and associated with long-term complications gravely affecting quality of life (fecal incontinence)^35,36^ Narrowing this outcome more would have resulted in even fewer events in each group, thus increasing risk for random errors and requiring another 20 years of data collection, while broadening it by including surrogate measures would dilute the importance of our findings. We included postpartum hemorrhage when diagnostic codes indicated a blood loss of at least 1000 ml, combined with codes for blood transfusion. We deemed that in Sweden, where transfusion policies are restrictive, this approach would capture only severe cases. Similarly, to focus only on severe adverse events, we chose not to include uterine rupture to the composite outcome, as the diagnostic codes for uterine rupture do not distinguish between clinically insignificant dehiscence and complete rupture. We considered severe cases of uterine rupture to be those associated with severe maternal or neonatal outcomes, which are already included in the composite outcome. To add detail, we also report each component of the main composite outcomes, as well as several other secondary outcomes and those included in the perinatal COS, which may facilitate readers’ own analyses or evidence synthesis.

The main limitation is the lack of information on planned delivery mode and indication for CS in any of the births, which is inherent to the medical birth register’s method of data collection. Therefore, some of the increased infant risk for the first vaginal breech could be due to unfavorable prerequisites for an unplanned breech birth. Likewise, the adverse outcomes for both the mother and infant in the first cesarean breech group could be overestimated since this group also included emergency cesarean deliveries. We are also aware that, despite composite outcome measures and a large sample size, the rarity of the studied outcomes in Sweden makes it difficult to detect statistically significant differences in severe outcomes and the confidence intervals of risk estimates are wide for some outcomes. Moreover, there were important differences between the exposed (first vaginal breech) and unexposed (first breech CS) women regarding background parameters, with more favorable characteristics in the exposed group, which could also affect the outcome of the second birth. Although adjustment for observed differences between the two groups or sensitivity analyses did not substantially change the risk estimates in the multivariable model, there could be residual unmeasured confounding from factors that are not present in the registers. For example, women with a first vaginal breech birth were likely selected towards a high probability of an uncomplicated second vaginal birth to an extent that we were unable to adjust for, for example a positive family history of childbirth, less anxiety, and generous pelvic measurements on pelvimetry prior to the first birth. Nota bene, we do not claim a causal inference between mode of first birth and the outcome but report the observed rates and risks. Another limitation of the generalizability of the results, is the liberal view on trial of labor after cesarean in Sweden. The proportion of women giving birth vaginally after breech CS was 65.4% in our study, which is relatively high but not unusual, compared to reported 30% in Pakistan, 56% in Ireland, and 72% in Finland^15–17^. Risk-benefit balance in settings with different approach to birth after CS could differ from our results, probably decreasing the risk for some adverse events (as for example uterine rupture), at the same time increasing risks for other adverse events (as for example major hemorrhage or hysterectomy).

The increased infant risk in the first birth as vaginal breech compared with the first birth as breech CS found in our study is in line with most previous reports^1,37,38^ Nevertheless, some studies report equal neonatal risks for vaginal breech birth compared with planned CS or with cephalic vaginal birth^39–41^. This discrepancy can possibly be explained by organizational factors or differences in intrapartum management rather than by populational characteristics or pregnancy complications^42–45^. For example, the overall rate of vaginal breech deliveries is relatively low in Sweden^46^, upright breech births are favored in Germany,^43,44^ and using forceps to deliver the aftercoming head in vaginal breech births is common in France and Norway^42,45,47^ In settings with a low rate of vaginal breech births, competence and skills to assist complicated vaginal breech births risk a rapid decline, which can lead to avoidance of vaginal breech birth, further decreasing the possibility to gain experience, and thus creating a vicious circle that can harm women and babies^1,48–51^ However, teaching may improve skills, expand obstetricians’ toolkit, and perhaps save the art of vaginal breech from extinction^43,52^.

The risk reduction for the second child of mothers with a first vaginal breech birth compared with those with a first breech CS is, despite the limitations above, in line with previous studies on vaginal birth after cesarean^7–10^. Most included births in previous studies are prior cephalic cesarean births, and hence the balance between maternal and infant outcomes are not equal to that of breech births. Most childbearing women opt for at least two children according to global fertility rates^53^, which motivates the calculation of a total “happy family” outcome: no severe adverse outcomes for the mother or any of the first two children. With the results of our study, a woman planning for two children may be counselled that a cesarean delivery for her first breech baby may reduce the risk for that child but increase the risk for her second child, giving a similar outcome for the two children in total. She herself will likely not experience more, or less, severe adverse outcomes with any mode of first birth. Theoretically, benefits of the first vaginal birth for the mother and her future children will be even more prominent if she will have more than two children, while risks associated with the first CS will increase with the number of children. With this information, clinicians may find it easier to support women’s autonomy and to be responsive to their values and preferences.

## Conclusion

The probability of a “happy family” avoiding severe outcomes for mother and her two first children whereof the first one was born in breech presentation was not significantly changed by mode of the first delivery in breech. Cesarean section for the first child in breech did not eliminate the risk of adverse infant outcome but rather moved it from the first child to the second. The maternal risks for any of the two births were similar regardless mode of the first breech birth. These findings shift risk-harm relations in breech presentation at or near term towards equilibrium and justify any choice of mode of breech birth as approximately equally reasonable in adequately selected cases and similar healthcare settings.

## Electronic supplementary material

Below is the link to the electronic supplementary material.

Supplementary Material 1

## Data Availability

This paper uses data from Statistics Sweden and the Swedish National Board of Health and Welfare. Because the data contain sensitive information on individuals, the Swedish law requires that users of the data obtain permission from the Swedish Ethical Review Authority.

## Abbreviations

aOR: Adjusted odds ratio
BMI: Body mass index
CI: Confidence interval
CNS: Central nervous system
CS: Cesarean section
ICD-10: International Classification of Diseases 10th edition
IVF: In vitro fertilization
HIE: Hypoxic ischemic encephalopathy
OASI: Obstetric anal sphincter injury
OR: Odds ratio
