# Supplementary material for: If the first child is breech, overall outcomes for families with two children are similar regardless of the mode of the first birth
Source: Sci Rep. 2024 Oct 16;14:24231. doi: 10.1038/s41598-024-76433-7 (PMC11484794; doi:10.1038/s41598-024-76433-7)
Supplement: Supplementary file 1 — Supplementary Material 1 [file 41598_2024_76433_MOESM1_ESM.pdf]

Supplementary material

Table S1. STROBE checklist of items that should be included in reports of ***cohort studies***

	Item No	Recommendation	Page
**Title and abstract**	1	(*a*) Indicate the study's design with a commonly used term in the title or the abstract	1
		(*b*) Provide in the abstract an informative and balanced summary of what was done and what was found	2
**Introduction**			
Background/rationale	2	Explain the scientific background and rationale for the investigation being reported	3-4
Objectives	3	State specific objectives, including any prespecified hypotheses	2,4
**Methods**			
Study design	4	Present key elements of study design early in the paper	1, 2, 4
Setting	5	Describe the setting, locations, and relevant dates, including periods of recruitment, exposure, follow-up, and data collection	4
Participants	6	(*a*) Give the eligibility criteria, and the sources and methods of selection of participants. Describe methods of follow-up	4-5
		(*b*) For matched studies, give matching criteria and number of exposed and unexposed	n/a
Variables	7	Clearly define all outcomes, exposures, predictors, potential confounders, and effect modifiers. Give diagnostic criteria, if applicable	2, 4-7
Data sources/ measurement	8*	For each variable of interest, give sources of data and details of methods of assessment (measurement). Describe comparability of assessment methods if there is more than one group	4-7
Bias	9	Describe any efforts to address potential sources of bias	4
Study size	10	Explain how the study size was arrived at	
Quantitative variables	11	Explain how quantitative variables were handled in the analyses. If applicable, describe which groupings were chosen and why	5-6
Statistical methods	12	(*a*) Describe all statistical methods, including those used to control for confounding	7
		(*b*) Describe any methods used to examine subgroups and interactions	7
		(*c*) Explain how missing data were addressed	6
		(*d*) If applicable, explain how loss to follow-up was addressed	n/a
		(*e*) Describe any sensitivity analyses	7
**Results**			
Participants	13*	(a) Report numbers of individuals at each stage of study—eg numbers potentially eligible, examined for eligibility, confirmed eligible, included in the study, completing follow-up, and analysed	8, Figure 2
		(b) Give reasons for non-participation at each stage	n/a
		(c) Consider use of a flow diagram	8, Figure 2
Descriptive data	14*	(a) Give characteristics of study participants (eg demographic, clinical, social) and information on exposures and potential confounders	8, Table1
		(b) Indicate number of participants with missing data for each variable of interest	8, Table 1
		(c) Summarise follow-up time (eg, average and total amount)	n/a
Outcome data	15*	Report numbers of outcome events or summary measures over time	8-9, Table 2-4
Main results	16	(*a*) Give unadjusted estimates and, if applicable, confounder-adjusted estimates and their precision (eg, 95% confidence	Table 2-4

		interval). Make clear which confounders were adjusted for and why they were included	
		(*b*) Report category boundaries when continuous variables were categorized	n/a
		(*c*) If relevant, consider translating estimates of relative risk into absolute risk for a meaningful time period	Table 2-4
Other analyses	17	Report other analyses done—eg analyses of subgroups and interactions, and sensitivity analyses	9
**Discussion**			
Key results	18	Summarise key results with reference to study objectives	9-10
Limitations	19	Discuss limitations of the study, taking into account sources of potential bias or imprecision. Discuss both direction and magnitude of any potential bias	10
Interpretation	20	Give a cautious overall interpretation of results considering objectives, limitations, multiplicity of analyses, results from similar studies, and other relevant evidence	11-12
Generalisability	21	Discuss the generalisability (external validity) of the study results	11
**Other information**			
Funding	22	Give the source of funding and the role of the funders for the present study and, if applicable, for the original study on which the present article is based	13

Table S2. Outcomes of the 1st breech birth, according to Swedish perinatal Core Outcome Set[1]

	1st birth		1st birth vaginal breech (n)	1st birth vaginal breech (%)	1st birth breech CS (n)	1st birth breech CS (%)	Total (n)	Total (%)
	Total		1525	100	21537	100	23062	100
1	Death, fetal or within 28 days after birth)	Antenatal stillbirth	n/a (excluded)	n/a	n/a (excluded)	n/a	n/a	n/a
		Intrapartal stillbirth	2	0.13	0	0.00	2	0.01
		Early neonatal death	9	0.59	9	0.04	18	0.08
		Late neonatal death	1	0.07	6	0.03	7	0.03
		Deaths in total	12	0.79	15	0.07	27	0.12
2	Vital at birth		1360	89.18	20856	96.84	22216	96.33
3	Undisturbed first time with mother		no data	no data	no data	no data	no data	no data
4	Neonatal intensive care unit (NICU) admission		120	7.87	960	4.46	1080	4.68
5	Serious neonatal morbidity within 28 days after birth		114	7.48	912	4.23	1026	4.45
6	Ongoing resuscitation (including assisted ventilation) initiated at birth and continued for at least 10 minutes		43	2.82	200	0.93	243	1.05
7	Apgar score of <6 at 10 minutes		8	0.52	9	0.04	17	0.07
8	Acidemia (pH <7.0 or a base deficit of ≥16 mmol/L in a sample of umbilical cord blood or neonatal blood obtained within the first hour after birth)		6	0.39	8	0.04	14	0.06
9	Need for therapeutic hypothermia		0	0.00	1	0.00	1	0.00
10	Hypoxic–ischemic encephalopathy (HIE), stratified by severity	Mild (HIE 1)	0	0.00	2	0.01	2	0.01
		Moderate (HIE 2)	1	0.07	3	0.01	4	0.02
		Severe (HIE 3)	0	0.00	0	0.00	0	0.00
		Unspecified	0	0.00	0	0.00	0	0.00
		HIE in total	1	0.07	5	0.03	7	0.03
11	Need for resuscitation AND metabolic acidosis (pH <7.0 and base deficit of ≥16 mmol/L in a sample of umbilical cord blood or neonatal blood obtained within the first hour after birth) AND following hypoxic–ischemic encephalopathy		0	0.00	3	0.01	3	0.0
12	Neonatal sepsis, confirmed by the culture from blood or sterile site		9	0.59	63	0.29	72	0.31
13	Breastfeeding at discharge		106	6.95	761	3.53	867	3.76
14	Birth by vaginal instrumental delivery because of fetal distress as one of the indications		16	1.05	0	0.00	16	0.07
15	Birth by cesarean section (CS) because of fetal distress as one of the indications		0	0.00	327	1.52	327	1.42
16	Gestational age (completed weeks of gestation)	22	n/a (excluded)	n/a	n/a (excluded)	n/a	n/a	n/a
		23	n/a (excluded)	n/a	n/a (excluded)	n/a	n/a	n/a
		24	n/a (excluded)	n/a	n/a (excluded)	n/a	n/a	n/a
		25	n/a (excluded)	n/a	n/a (excluded)	n/a	n/a	n/a
		26	n/a (excluded)	n/a	n/a (excluded)	n/a	n/a	n/a
		27	n/a (excluded)	n/a	n/a (excluded)	n/a	n/a	n/a
		28	n/a (excluded)	n/a	n/a (excluded)	n/a	n/a	n/a
		29	n/a (excluded)	n/a	n/a (excluded)	n/a	n/a	n/a

	1st birth		1st birth vaginal breech (n)	1st birth vaginal breech (%)	1st birth breech CS (n)	1st birth breech CS (%)	Total (n)	Total (%)
		30	n/a (excluded)	n/a	n/a (excluded)	n/a	n/a	n/a
		31	n/a (excluded)	n/a	n/a (excluded)	n/a	n/a	n/a
		32	n/a (excluded)	n/a	n/a (excluded)	n/a	n/a	n/a
		33	n/a (excluded)	n/a	n/a (excluded)	n/a	n/a	n/a
		34	56	3.67	411	1.91	467	2.02
		35	83	5.44	669	3.11	752	3.26
		36	118	7.74	1043	4.84	1161	5.03
		37	137	8.98	2031	9.43	2168	9.40
		38	242	15.87	8338	38.71	8580	37.20
		39	339	22.23	6464	30.01	6803	29.50
		40	351	23.02	1515	7.03	1866	8.09
		41	166	10.89	798	3.71	964	4.18
		42	33	2.16	261	1.21	294	1.27
		> 42	0	0.00	7	0.03	7	0.03
17	Birthweight, grams	< 500	4	0.26	19	0.09	23	0.10
		500 - 999	1	0.07	0	0.00	1	0.00
		1000 - 1499	1	0.07	16	0.07	17	0.07
		1500 - 1999	12	0.79	191	0.89	203	0.88
		2000 - 2499	126	8.26	1135	5.27	1261	5.47
		2500 - 2999	423	27.74	4575	21.24	4998	21.67
		3000 - 3499	658	43.15	8862	41.15	9520	41.28
		3500 - 3999	262	17.18	5281	24.52	5543	24.04
		4000 - 4499	38	2.49	1254	5.82	1292	5.60
		4500 - 4999	0	0.00	180	0.84	180	0.78
		5000 and more	0	0.00	24	0.11	24	0.10

Table S3. Outcomes of the 2nd birth following 1st breech birth, according to Swedish perinatal Core Outcome Set[1]

	2nd birth (women with 1st birth in breech)		Previous breech vaginal birth (n)	Previous breech vaginal birth (%)	Previous breech CS (n)	Previous breech CS (%)	Total (n)	Total (%)
	Total		1525	100	21537	100	23062	100
1	Death, fetal or within 28 days after birth)	Antenatal stillbirth	1	0.1	51	0.2	52	0.23
		Intrapartal stillbirth	0	0.00	9	0.04	9	0.04
		Early neonatal death	1	0.07	21	0.10	22	0.10
		Late neonatal death	0	0.00	6	0.03	6	0.03
		Deaths in total	2	0.13	87	0.40	89	0.39
2	Vital at birth		1497	98.2	20619	95.7	22116	95.9
3	Undisturbed first time with mother		no data	no data	no data	no data	no data	no data
4	Neonatal intensive care unit (NICU) admission		68	4.46	1189	5.52	1257	5.45
5	Serious neonatal morbidity within 28 days after birth		61	4.00	1106	5.14	1167	5.06
6	Ongoing resuscitation (including assisted ventilation) initiated at birth and continued for at least 10 minutes		13	0.85	309	1.43	322	1.40
7	Apgar score of <6 at 10 minutes		0	0.00	45	0.21	45	0.2
8	Acidemia (pH <7.0 or a base deficit of ≥16 mmol/L in a sample of umbilical cord blood or neonatal blood obtained within the first hour after birth)		0	0.00	83	0.39	83	0.36
9	Need for therapeutic hypothermia		0	0.00	22	0.10	22	0.10
10	Hypoxic–ischemic encephalopathy (HIE), stratified by severity	Mild (HIE 1)	0	0.00	13	0.06	13	0.06
		Moderate (HIE 2)	0	0.00	16	0.07	16	0.07
		Severe (HIE 3)	0	0.00	7	0.03	7	0.03
		Unspecified	0	0.00	0	0.00	0	0.00
		HIE in total	0	0.00	36	0.17	36	0.16
11	Need for resuscitation AND metabolic acidosis (pH <7.0 and base deficit of ≥16 mmol/L in a sample of umbilical cord blood or neonatal blood obtained within the first hour after birth) AND following hypoxic–ischemic encephalopathy		0	0.00	31	0.14	31	0.13
12	Neonatal sepsis, confirmed by the culture from blood or sterile site		4	0.26	146	0.68	150	0.65
13	Breastfeeding at discharge		54	3.54	940	4.36	994	4.31
14	Birth by vaginal instrumental delivery because of fetal distress as one of the indications		13	0.85	995	4.62	1008	4.37
15	Birth by cesarean section (CS) because of fetal distress as one of the indications		9	0.59	868	4.03	877	3.80
16	Gestational age (completed weeks of gestation)	22	0	0.00	4	0.02	4	0.02
		23	0	0.00	7	0.03	7	0.03
		24	0	0.00	12	0.06	12	0.05
		25	0	0.00	14	0.07	14	0.06
		26	1	0.07	6	0.03	7	0.03
		27	0	0.00	11	0.05	11	0.05
		28	2	0.13	13	0.06	15	0.07
		29	1	0.07	21	0.10	22	0.10
		30	0	0.00	27	0.13	27	0.12
		31	1	0.07	37	0.17	38	0.16

	2nd birth (women with 1st birth in breech)		Previous breech vaginal birth (n)	Previous breech vaginal birth (%)	Previous breech CS (n)	Previous breech CS (%)	Total (n)	Total (%)
		32	5	0.33	50	0.23	55	0.24
		33	7	0.46	88	0.41	95	0.41
		34	11	0.72	120	0.56	131	0.57
		35	26	1.70	249	1.16	275	1.19
		36	44	2.89	482	2.24	526	2.28
		37	111	7.28	1166	5.41	1277	5.54
		38	268	17.57	4019	18.66	4287	18.59
		39	443	29.05	5420	25.17	5863	25.42
		40	395	25.90	5101	23.68	5496	23.83
		41	170	11.15	3348	15.55	3518	15.25
		42	37	2.43	1294	6.01	1331	5.77
		> 42	3	0.20	42	0.20	45	0.20
17	Birthweight, grams	< 500	0	0.00	7	0.03	7	0.03
		500 - 999	0	0.00	47	0.22	47	0.20
		1000 - 1499	4	0.26	58	0.27	62	0.27
		1500 - 1999	5	0.33	127	0.59	132	0.57
		2000 - 2499	38	2.49	438	2.03	476	2.06
		2500 - 2999	203	13.31	2342	10.87	2545	11.04
		3000 - 3499	540	35.41	7132	33.12	7672	33.27
		3500 - 3999	517	33.90	7520	34.92	8037	34.85
		4000 - 4499	181	11.87	3087	14.33	3268	14.17
		4500 - 4999	31	2.03	634	2.94	665	2.88
		5000 and more	4	0.26	122	0.57	126	0.55

1. Savchenko J, Asp M, Blomberg M, Elvander C, Hagman A, Pegelow Halvorsen C, et al. Key outcomes in childbirth: Development of a perinatal core outcome set for management of labor and delivery at or near term. Acta Obstet Gynecol Scand. 2023 Jun;102(6):728-34.
